# The Acute Effects of Time-Varying Caloric Vestibular Stimulation as Assessed With fMRI

**DOI:** 10.3389/fnsys.2021.648928

**Published:** 2021-08-09

**Authors:** Robert D. Black, Ryan P. Bell, Kristal M. Riska, Christopher Spankovich, Richard W. Peters, Christopher D. Lascola, Christopher T. Whitlow

**Affiliations:** ^1^Scion NeuroStim, LLC, Durham, NC, United States; ^2^Department of Psychiatry & Behavioral Sciences, Duke University School of Medicine, Durham, NC, United States; ^3^Department of Head and Neck Surgery & Communication Sciences, Duke University School of Medicine, Durham, NC, United States; ^4^Department of Otolaryngology & Head and Neck Surgery, University of Mississippi Medical Center, Jackson, MS, United States; ^5^Department of Radiology and Neurobiology, Duke University School of Medicine, Durham, NC, United States; ^6^Department of Radiology, Wake Forest School of Medicine, Winston-Salem, NC, United States

**Keywords:** caloric vestibular stimulation, sensory neuromodulation, non-invasive neuromodulation device, BOLD imaging, independent component analysis, vestibular system

## Abstract

We describe preliminary results from the application of time-varying caloric vestibular stimulation (tvCVS) to volunteers during a continuous blood oxygen level dependent (BOLD) functional MRI (fMRI) acquisition, recording baseline, during-tvCVS and post-tvCVS epochs. The modifications necessary to enable the use of this novel device in a 3-Tesla magnetic field are discussed. Independent component analysis (ICA) was used as a model-free method to highlight spatially and temporally coherent brain networks. The ICA results are consistent with tvCVS induction being mediated principally by thermoconvection in the vestibular labyrinth and not by direct thermal effects. The activation of hub networks identified by ICA is consistent with the concept of sensory neuromodulation, which posits that a modulatory signal introduced to a sensory organ is able to traverse the regions innervated (directly and indirectly) by that organ, while being transformed so as to be “matched” to regional neuronal dynamics. The data suggest that regional neurovascular coupling and a systemic cerebral blood flow component account for the BOLD contrast observed. The ability to modulate cerebral hemodynamics is of significant interest. The implications of these initial findings for the use of tvCVS therapeutically are discussed.

## Introduction

Non-invasive neuromodulation devices (NIND’s) are being evaluated for use in treating neurological disease (Bain et al., 2015). A subset of NIND’s focus on stimulation of cranial nerves (Adair et al., 2020) in order to purposefully modulate an endogenous network, versus a narrowly defined target in the brain stimulated by an implanted electrode (e.g., deep brain stimulation, DBS) or methods like transcranial magnetic stimulation (TMS) and transcranial direct or alternating current stimulation (tDCS, tACS) that apply currents to large areas of the brain, primarily in the neocortex (Danilov et al., 2015). Devices that modulate the 8th cranial nerve, the vestibulocochlear, are of two primary types: galvanic (GVS) and caloric (CVS). The former, GVS, has been used in numerous research studies (Fitzpatrick and Day, 2004 *and references therein*). It is generally accomplished by applying direct or alternating current to the electrodes placed on the mastoid bones behind the ears. This creates a voltage bias between the right and left side vestibular organs. The latter, caloric vestibular stimulation, is best known as a diagnostic method used in balance clinics (Fitzgerald and Hallpike, 1942). CVS involves irrigating the external auditory canal with water or air, above or below body temperature. The thermal gradient leads to altered firing rates of the vestibular hair cells. Recently, a device that delivers time-varying CVS (tvCVS) has been developed to explore the potential of this form of cranial nerve neuromodulation for therapeutic purposes (Black et al., 2016). Time variation of the thermal stimulus avoids adaptation to the stimulus that will occur with a constant temperature stimulus and is crucial for delivering CVS over extended periods of time. Controlling the time-rate-of-change of temperature also mitigates side effects that can be produced by rapid changes in temperature during diagnostic CVS procedures.

Our central aim is to provide preliminary, proof-of-concept results from a study evaluating acute effects of tvCVS as measured with BOLD (blood oxygen level dependent) functional MRI (fMRI). This work was motivated by the observation of the induction of cerebral blood flow velocity (CBFv) oscillations with tvCVS in prior work (Black et al., 2016). That earlier study was based on the insonation of the basilar artery and therefore did not provide any details on which brain regions were modulated. By running a continuous (40-min long) BOLD scan we were able to gather data during a baseline period, during the time tvCVS was being delivered and for a period after the device was turned off again. The organization of this report is as follows: (1) We start with a review of cerebral autoregulation and spontaneous flow oscillations in order to provide context for the observation of low frequency CBFv oscillations induced by tvCVS. (2) Next we provide a short overview of the study of spontaneous low frequency oscillations in BOLD imaging. (3) We then describe the means by which the tvCVS device was integrated into the MRI environment and discuss the particular image analysis methods used. (4) We discuss how induced tvCVS effects seem to propagate through the vestibular system in a way that is consistent with the concept of sensory neuromodulation (Black and Rogers, 2020) and how that model might explain clinical tvCVS study data. (5) Finally, we talk about how the results from this exploratory study might inform future work.

## Background

It is important to highlight a finding that has been reported in multiple studies involving vestibular stimulation: cyclic modulation of systemic and cerebral blood flow. The creation of CBFv oscillations by tvCVS, as described below, may be important in explaining clinical results that have been reported using this method. Additionally, it is important to review past fMRI studies that characterize cerebral blood flow changes, oscillatory behavior in particular, so that the effects of tvCVS on flow can be put into context.

### Hemodynamic Oscillations—Integral to Brain Function

Cerebral autoregulation is well-studied and is recognized as being fundamental for maintaining dynamic brain function (McHenry et al., 1974; Strandgaard and Paulson, 1984; Giller, 1990; Flammer and Mozaffarieh, 2008; Reinhard et al., 2008; Greene and Lee, 2012; Horsfield et al., 2013). Unsurprisingly, dysfunction of cerebral autoregulation has been noted in patients with neurological disease (Czosnyka et al., 2001; Eames et al., 2002; Haubrich et al., 2003; Claassen and Zhang, 2011; Gommer et al., 2012; Medow et al., 2014; Shekhar et al., 2017), but not necessarily as a consequence of normal aging (Carey et al., 2000). Cerebral autoregulation is a dynamic phenomenon and encompasses a range of processes, including so-called B wave oscillations and neurovascular coupling (NVC), as described below.

Time-varying caloric vestibular stimulation was shown, using transcranial Doppler sonography, to be capable of inducing oscillations in pulsatility index and CBFv in the so-called B wave frequency range, which is broadly defined at 0.5–3.0 cycles per minute or 0.008–0.05 Hz (Lundberg, 1960). B waves may play an important role in dynamic autoregulation (Lemaire et al., 2002), representing a characteristic frequency range relevant to successful homeostatic control and illustrating the relevance of hemodynamic oscillations in achieving such control. Previously, the effects of vestibular stimulation on CBFv have been reported. Constant temperature CVS (Tiecks et al., 1996; Heckmann et al., 1999) was found to alter (reduce or increase) CBFv in major cerebral arteries. Oscillations in CBFv were not induced, however, because the application of constant temperature CVS creates a unidirectional change in flow rate. Serrador et al. (2009) invoked vestibular responses by moving subjects with a high-torque hydraulic-powered tilt chair and observed corresponding oscillations in CBFv (middle cerebral artery) and blood pressure (BP). The tilt chair was actuated in a frequency range between 0.03125 and 0.5 Hz. The CBFv and BP changes seemed to have been induced with little or no time lag once the chair started moving. The authors proposed two pathways from the vestibular nuclei to effects on cerebral vessels. The first pathway was through the nucleus tractus solitarius. The second was via the fastigial nucleus in the cerebellum, which is a pathway studied by Reis et al. (1991). [It is worth noting that Reis and collaborators extensively researched electrical stimulation of the fastigial nucleus as a means of providing neuroprotection (Glickstein et al., 2003; Zhou et al., 2005; Golanov et al., 2017)]. Cohen et al. (2011) used sinusoidal GVS to induce vasovagal responses in a rat model. The authors measured sinusoidal heart rate and BP changes resulting from the application of sinusoidal GVS over a frequency range of 0.008–0.5 Hz, thus very similar to the range used by Serrador et al. (2009). GVS is thought to act primarily via irregular afferents (Goldberg et al., 1984), which constitute roughly 25% of the total population (regular afferents comprising the balance). CVS, by comparison, is capable of stimulating both regular and irregular afferents.

Taken together, these results show that time-varying vestibular stimulation in the B wave frequency range can result in induced oscillations in hemodynamic variables, including CBFv. We have consciously emphasized the range of frequencies covered by the “B wave” definition in part because that term does not typically appear in imaging studies of slow wave oscillations in neuronal activity in the brain. Our goal is to argue that systemic blood flow oscillations and oscillations in neural network activity need to be considered in concert since even if they arise from independent mechanisms, overlaps in frequency can lead to mutual interactions (oscillatory coupling). For example, cerebral autoregulation, generally viewed as a systemic process, ensures that NVC, a localized process, can function properly (Azevedo et al., 2007; Fritzsch et al., 2010).

### Slow BOLD Oscillations

Slow oscillations in BOLD signal have been observed in resting state fMRI studies and understanding their origins is an ongoing area of research. Studies of the default mode network are typically focused on the frequency range ∼0.01–0.1 Hz (Zuo et al., 2010; Lv et al., 2018). Changes in resting state functional connectivity, the term given to measurements of temporal correlations within this frequency range, have proven to be a productive tool in analyzing changes in the default mode network as a function of neurological disease (see Raichle et al., 2001; Raichle, 2015). Recently Tong et al. (2019) analyzed contributions to BOLD oscillations in the ∼0.01–0.15 Hz range and provided evidence that in addition to a signal component generated by neuronal activity via NVC, there was a systemic component due to blood flow changes responsible for at least 30% of the signal seen in gray matter. Tong et al. (2019) refer to this as a non-neuronal signal when, more particularly, they mean a signal that was not caused by regional NVC (ultimately, the flow changes will of course have a central and/or peripheral neuronal interface). The systemic signal component was found, as expected, to have a phase lag between anatomically separate regions due to the finite speed of the flow changes (Murphy et al., 2013; Aso et al., 2017). Proposals for the origins of NVC-mediated slow oscillations include alterations in autonomic tone, fluctuations in the partial pressure of CO_2_, blood pressure regulation, vasomotor oscillations, low frequency “neuronal waves,” and gastric motility (Tong et al., 2019; Drew et al., 2020).

Are the slow frequency oscillations reported in BOLD resting-state studies related to slow oscillations in CBFv? Whittaker et al. (2019) consider the possible links between dynamic cerebral autoregulation and low frequency oscillations in resting-state fMRI. At this time, it’s not possible to provide a detailed answer about causality and mechanistic origins of all of the different neuronal and hemodynamic processes that have been discussed as giving rise to low frequency oscillations. However, as previously stated, even if oscillations arise from different origins, they can interact due to the overlap of their frequency ranges (Buzsáki, 2006; Gohel and Biswal, 2015; Black and Rogers, 2020). For example, a non-local CBFv oscillatory process will interact with a regional NVC-driven oscillatory process since both affect flow and blood vessel compliance concurrently. In this regard, they should not be viewed as truly independent processes since their effects can feed back on each other.

To see how previously observed oscillations in CBFv might be manifested in an imaging context, we performed a preliminary fMRI study, delivering tvCVS to four volunteers during a continuous BOLD acquisition.

## Materials and Methods

### tvCVS Used With MRI

**FIGURE 1:**
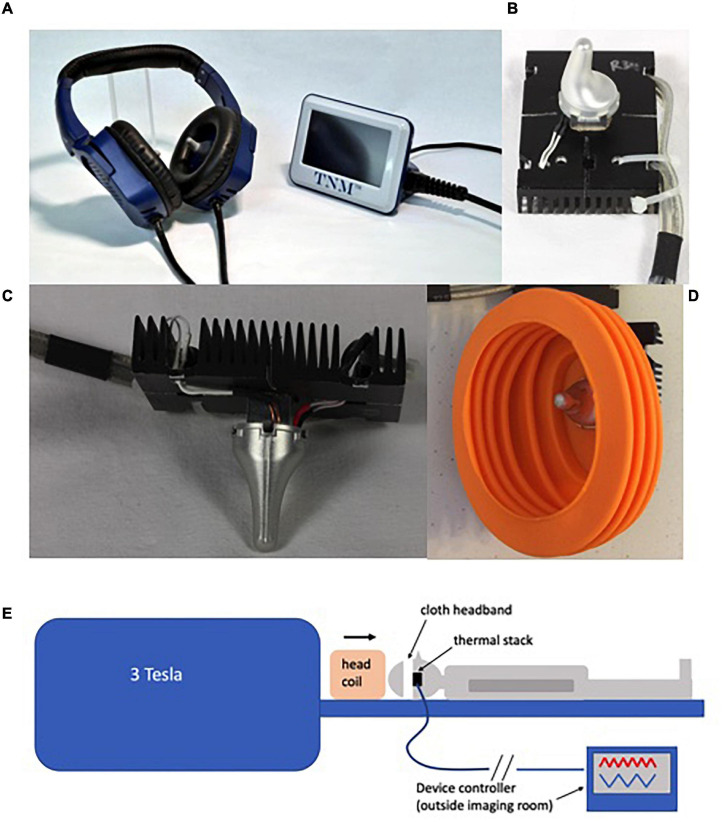
**(A)** A tvCVS device (Scion NeuroStim, Durham, NC); **(B)** The earpiece/Peltier device/heat sink thermal stack; **(C)** Side view of the thermal stack; **(D)** A baffle used to reduce acoustic noise during the BOLD scan; **(E)** A schematic showing the environment of the imaging runs.

A closed-cycle CVS device, using circulating water at a *constant* temperature, has previously been developed for MRI studies (Frank and Greenlee, 2014). However, for the current study, it was important to use the same *time-varying* CVS method that has been used in clinical trials of migraine headache (Wilkinson et al., 2017) and Parkinson’s disease (Wilkinson et al., 2019). A standard device headset (Figure 1) has ferrous metallic hardware and cooling fans, making it unsuitable for use in a strong magnetic field. To accommodate the tvCVS device to an MRI setting it was modified to remove ferromagnetic components and reduce eddy currents generated by the aluminum heat sink, the largest remaining metallic element. Initial tests showed significant image artifacts created by the heat sinks and so cuts were made from the edges of the heat sink to the center (Figure 1B) in order to shrink the diameter of induced eddy currents that create susceptibility artifacts. Other components of this modified device that are subjected to the magnetic field are the aluminum earpiece, a Peltier thermoelectric cooler and a thermistor (temperature varying resistor). These components are secured together into one assembly with thermally conductive epoxy. This assembly is referred to as the thermal stack. In a standard headset, the thermal stack also consists of a printed circuit board that controls the current to the Peltier device and monitors the resistance of the thermistor. On the modified device, a cable 25 feet in length was used to connect the thermal stack to the printed circuit board, allowing the device electronics to be positioned outside the MRI scanner room. The individual conductors in the cable are coaxial with a signal wire surrounded by a braided shield to reduce noise induced into the signal wires by time-varying magnetic fields. To further reduce noise that could potentially be induced into the signal wires of the cable, low pass filters were added to the signal inputs on the printed circuit board. Additionally, transient voltage suppressors were added to each signal input to protect electronic circuits from voltage spikes induced into the cable by the magnetic field gradients. The amount of noise introduced into the cable by the magnetic field gradients of the MRI scanner was measured during initial testing to confirm the cable shielding, input filtering and voltage suppression limited the size of induced noise. A rubber baffle (Figure 1D) was added to reduce the amount of scanner noise experienced by the subjects. The tvCVS devices were held in place on subjects’ heads by an elastic cloth band (Figure 1E) prior to the insertion of the subject’s head into a head coil.

**FIGURE 2:**
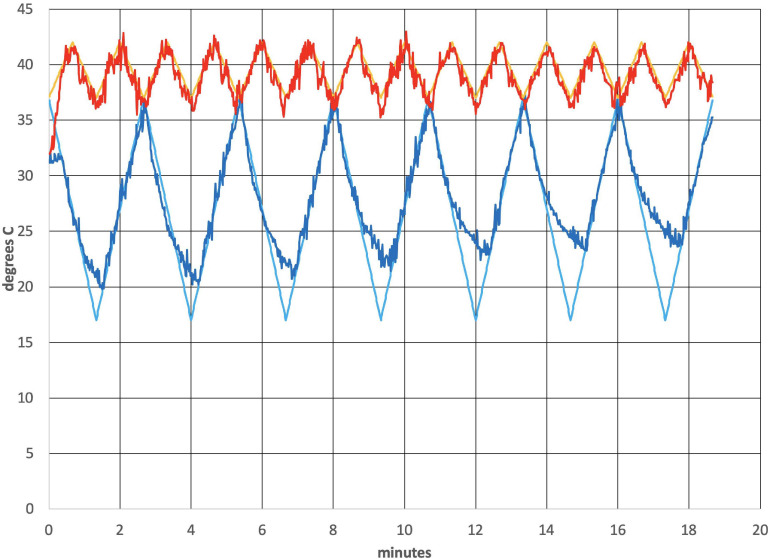
Temperature profile from an actual run (subject 4). All subjects had L-ear cold stimulation. The smooth lines are the target temperature profiles.

Figure 2 shows the thermal waveforms measured during an actual run. Two simultaneous thermal waveforms were delivered: a cold triangular pattern that went from 37 to 17 °C with a period of 2.67 min and a warm triangular pattern that went from 37 to 42°C with a period of 1.33 min (exactly ½ of the cold period). Thermistors in the earpieces measure the actual temperature and those data are overlaid on the target temperature values. There is some noise evident in the tracking of actual temperature to the target values. It is also apparent that as the run progressed, the ability of the cold waveform to reach 17°C, the target value (smooth blue line in Figure 2), was reduced. The mismatch is due to a rise in the heat sink temperature, which is exacerbated by the removal of the cooling fans that are normally present and by the elevated temperature in the bore of the magnet. This particular waveform combination was chosen to be consistent with previous clinical studies using the tvCVS device (Wilkinson et al., 2017, 2019).

### Subjects and Image Acquisition

**FIGURE 3:**
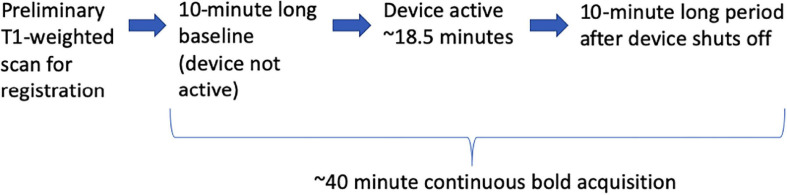
The sequence of events during the BOLD runs.

A study protocol entitled “BOLD fMRI Study of Cerebral Blood Flow Patterns Associated with the Induction of Time-Varying Caloric Vestibular Stimulation” was approved by the Wake Forest Baptist Medical Center Independent Review Board (IRB 00055459). Four volunteer subjects provided informed consent. All subjects were right-handed. The acquisition sequence is listed in Figure 3. No adverse events were reported by the subjects. Each subject was imaged once.

Data were acquired on a 3.0T Siemens Skyra whole-body scanner using a 32 channel, 9-inch diameter head coil. High-resolution T1-weighted (T1w) images were recorded using MPRAGE-GRAPPA2 sequence [repetition time (TR) = 2,300 ms, echo time (TE) = 3 ms, field of view (FOV) = 240 × 256, 240 × 240 matrix, 9° flip angle, interleaved slices of 1mm thickness, 192 slices]. Whole-brain BOLD images were collected using T2^∗^-weighted echo-planar imaging with the following parameters: TR = 2,000 ms; in-plane matrix size = 96 × 104; slice thickness = 2 mm; FOV = 864 × 936, voxel size of 2 mm × 2 mm × 2 mm, TE = 30 ms, flip angle = 52°, 72 slices.

Independent component analysis (ICA) was used to enable a model-free exploration of the BOLD data (Beckmann and Smith, 2004). ICA identified brain networks that exhibited both spatial and temporal coherence and provided the principal framework for our analyses. It is important to note that not all regions or all subjects generated the spatial and temporal signal coherence needed to be identified using ICA, but this does not necessarily mean that BOLD contrast resulting from time-varying CVS induction was absent. That is, incoherent BOLD signals would not be identified by ICA. We found it to be easier to present an unbiased report on these preliminary data by focusing first on model-free ICA networks. We specified that 30 independent components should be selected, ensuring that each subject would have the same number of components. This number of components has commonly been utilized for analyses utilizing Multivariate Exploratory Linear Optimized Decomposition into Independent Components (MELODIC). ICA traces in the Results section follow the baseline, tvCVS induction and post-tvCVS periods. The y-axis of the ICA plots shows the standard score (also called z-value), which is the number of standard deviations by which a value is above or below the mean value. Y-values above the mean have positive standard scores, while those below the mean have negative standard scores. Conceptually, the y-axis values represent BOLD signal magnitude. The results of the ICA analysis were then used to inform follow-up analyses. After conducting the ICA analyses, we were interested in how multimodal processing regions responded to tvCVS. BOLD images using anatomic masks were created for the cerebellum, hippocampus, thalamus and precuneous. Finally, we investigated neural activation in a frontoparietal network during the baseline, device-active and device-off time periods so as to better understand how tvCVS influences higher-order neural activation.

## Results

**FIGURE 4:**
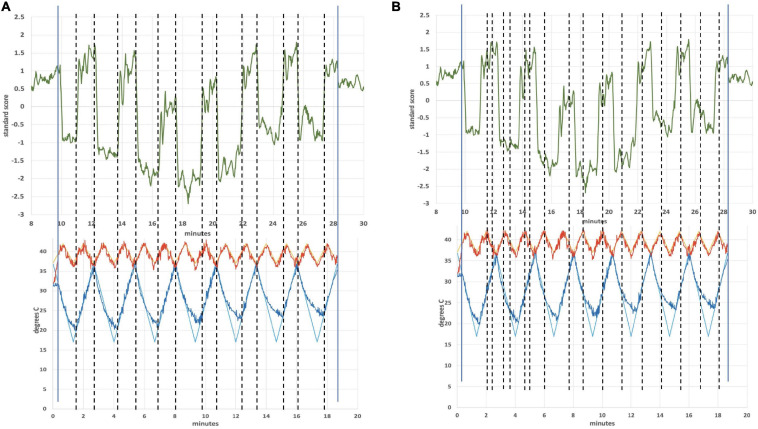
**(A)** Cerebellar independent component, subject 4, overlaid with the thermal waveforms. The dotted lines are drawn to match features in the cold waveform to the ICA trace. For example, the two leftmost dotted lines are aligned with the first low point of the cold waveform and the subsequent high point. Those dotted lines align with the transitions between plateaus and troughs in the ICA trace; **(B)** Cerebellar independent component, subject 4, highlighting the warm waveform. The dotted lines match features in the warm waveform to the ICA trace, suggesting that the warm waveform may cause additional structure in the plateaus and troughs, even though the cold waveform creates the dominant effect on the ICA trace.

Figure 4 is an ICA response from subject 4, identified as originating from the cerebellum. The Harvard-Oxford cortical and subcortical structural atlas (part of the FSL software package used for analyses) was utilized to identify neuroanatomical regions within the functional connectivity networks. Specifically, the functional connectivity networks were visualized using FSLview and the Harvard-Oxford cortical and subcortical structural atlas was used to pinpoint neuroanatomical regions with a minimum probability of 90%. The subject’s corresponding time-varying CVS waveform is superimposed, highlighting the cold pattern (applied to the left ear) in Figure 4A. The relative starting times for the ICA response and the thermal waveforms can’t be known precisely. We drew dotted lines corresponding to the high and low temperature values (again, for the cold waveform in Figure 4A) and then stretched the ICA trace to provide the best overlap with its features. That *all* of the dotted lines match thermal waveform features to ICA features so well suggests a stimulus-response relationship. As the run progresses, the width (time) of the cooling phase of the waveform lengthens and the warming phase shortens. This developing asymmetry as the run progresses is reflected in the ICA pattern. In the section “Discussion” we will present a hypothesis as to why the triangular thermal stimulus pattern results in a square wave ICA response.

The plateaus (relatively flat regions in Figure 4 with higher y-axis values) and troughs (relatively flat regions in Figure 4 with lower y-axis values) represent higher and lower, respectively, BOLD intensity, which is proportional to neuronal firing rate (firing rate of the vestibular hair cells). As described in the Discussion section, warm CVS increases hair cell firing rate and cold CVS decreases the firing rate. Therefore, plateaus correspond to faster firing (warming) and troughs correspond to slower firing (cooling). The plateaus and troughs of the cerebellar independent component square wave pattern have additional features suggesting a possible perturbation, perhaps due to the warm waveform (applied simultaneously to the right ear). Figure 4B shows dotted lines connecting features in the warm waveform to corresponding features in the cerebellar independent component. As in Figure 4A, alignment of the two traces was accomplished by stretching the ICA trace to provide the best overlap with its features. There appears to be alignment between the features of the warm waveform where a slope change occurs and points of inflection in the independent component trace. We are not able to prove that there is a causal correlation between the slope changes in the warm waveform and inflection points in the ICA trace, however, a hypothesis for the correlation is presented in the section “Discussion.”

Based on the square-wave appearance of the ICA time series, we decided to explicitly model the plateau periods and the trough periods during the device-on condition to further explore cerebellar activation in subject 4 using a block design (see Supplementary Material). We also modeled the plateaus and troughs of the square wave in the other three subjects to see if similar neural activations were present in the cerebellum. Subject 4 had an exceptionally strong cerebellar ICA response and our goal was to assess whether the other subjects had consistent, albeit smaller, responses. Specifically, we utilized an anatomic mask of the cerebellum and performed a block experimental design procedure, average across the other three subjects.

**FIGURE 5:**
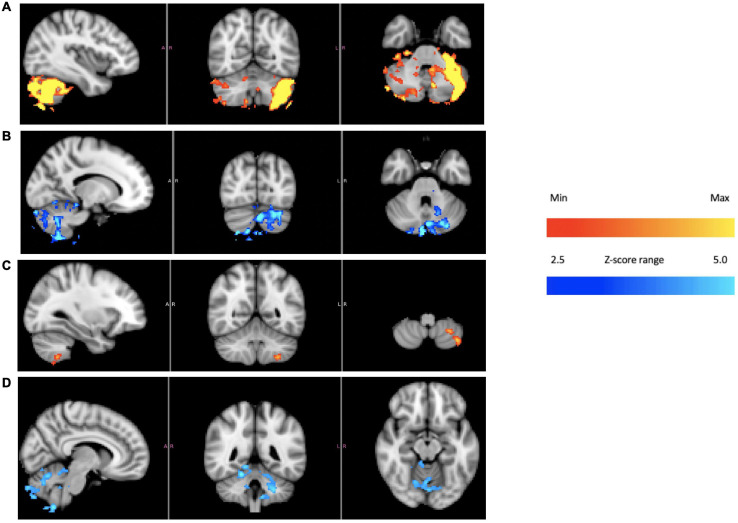
Cerebellum of subject 4 [**(A)** plateaus dominant, **(B)** troughs dominant]; cerebellum of subjects 1–3 averaged together [**(C)** plateaus dominant, **(D)** troughs dominant]. The color bars show relative z-score values. The orange/red and blue/aqua color bars are *not* related to each other.

Figure 5 shows BOLD intensity images reconstructed to emphasize the contrast for subject 4 due to the plateaus dominant (Figure 5A) and troughs dominant (Figure 5B), individually, based on the square wave pattern seen in Figure 4 (from the ICA analysis). Different regions in the cerebellum respond to reduced hair cell firing rate, troughs dominant, and increased firing rate, plateaus dominant (Supplementary Material). As noted, the other three subjects did not show a similarly large signal-to-noise ICA response in the cerebellum and it is not wholly clear why subject 4 was a “super responder” (see section “Discussion”). However, using the fit to the ICA pattern from subject 4 to model the BOLD image data from subjects 1–3 produced Figures 5C,D. The images from subject 4 alone and the averaged images from subjects 1–3 display a qualitatively similar pattern, in particular BOLD intensity lateralized to the left posterior cerebellum for the plateaus dominant state and a less lateralized, more central intensity pattern for the troughs dominant state.

**FIGURE 6:**
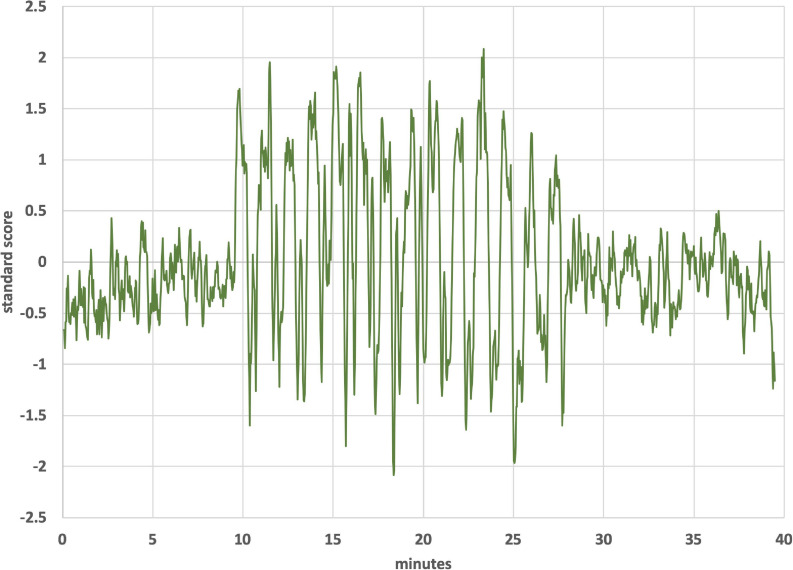
Independent component from operculum of subject 1. The trace was smoothed by averaging each point with 4 nearest neighbors.

**FIGURE 7:**
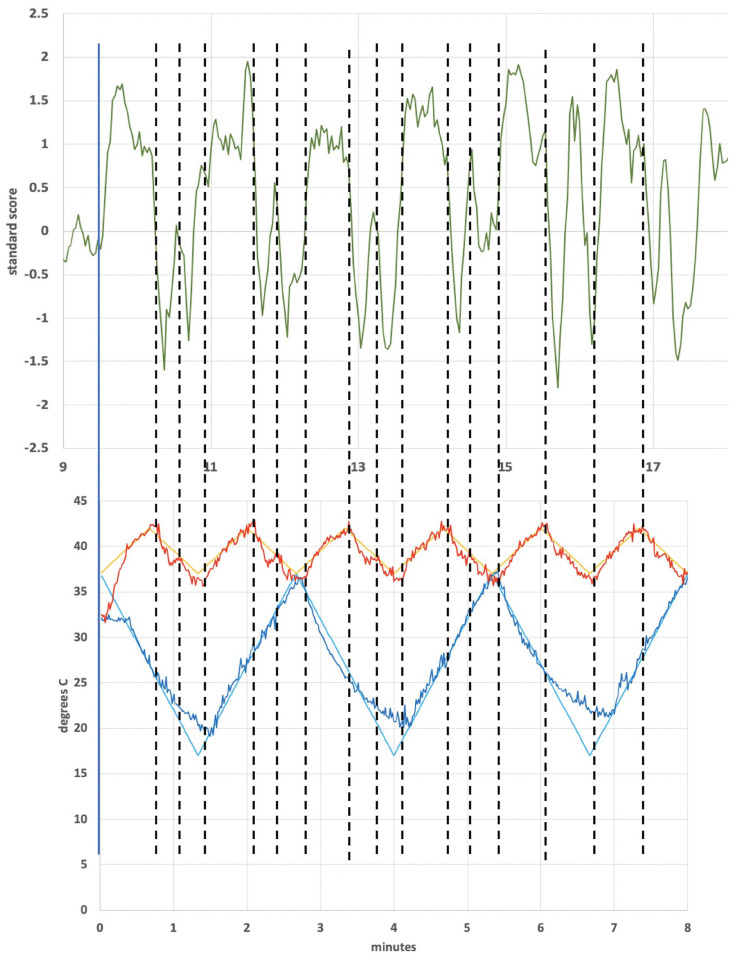
Subject 1 warm waveform (first 6 cycles) and independent component localized in the operculum. The dotted lines match features in the warm waveform to the ICA trace. For example, the leftmost and third-from-left dotted line align with a high point on the warm waveform and a low point, respectively. These lines align with a trough in the ICA trace. The second-from-left dotted line was added to highlight a small, retrograde feature in the warm waveform that aligns with a peaked feature in the ICA.

Figure 6 shows an independent component from subject 1 that lies in the operculum. The operculum is a multimodal processing region and so would be more closely identified with second-order sensory processing. It also encompasses the parieto-insular vestibular cortex (Frank and Greenlee, 2018). Unlike the case for the cerebellar independent component of subject 4, the period of which was dominated by the cold waveform period, the period the independent component from the operculum of subject 1 is dominated by the warm waveform period. Figure 7 shows the alignment of features in the warm waveform with corresponding features in the independent component. The low and high temperature points in the warm waveform correspond to sharp vertical edges in the independent component, as before with subject 4. But it also appears that features in the warm waveform that appear due to imprecise tracking control give rise to smaller plateaus in the independent component. We present a hypothesis that may explain this behavior in the section “Discussion.”

To understand better how multimodal processing regions may have responded during the plateau and trough periods, we examined activation in known hub regions including the thalamus, hippocampus and precuneous. This was accomplished by utilizing anatomic masks of these regions and performing a block experimental design, averaging across subjects. Finally, we identified the ICA of a frontoparietal network and modeled the baseline, device-on and device-off time periods to examine the character of neural activation, averaging across all subjects.

**FIGURE 8:**
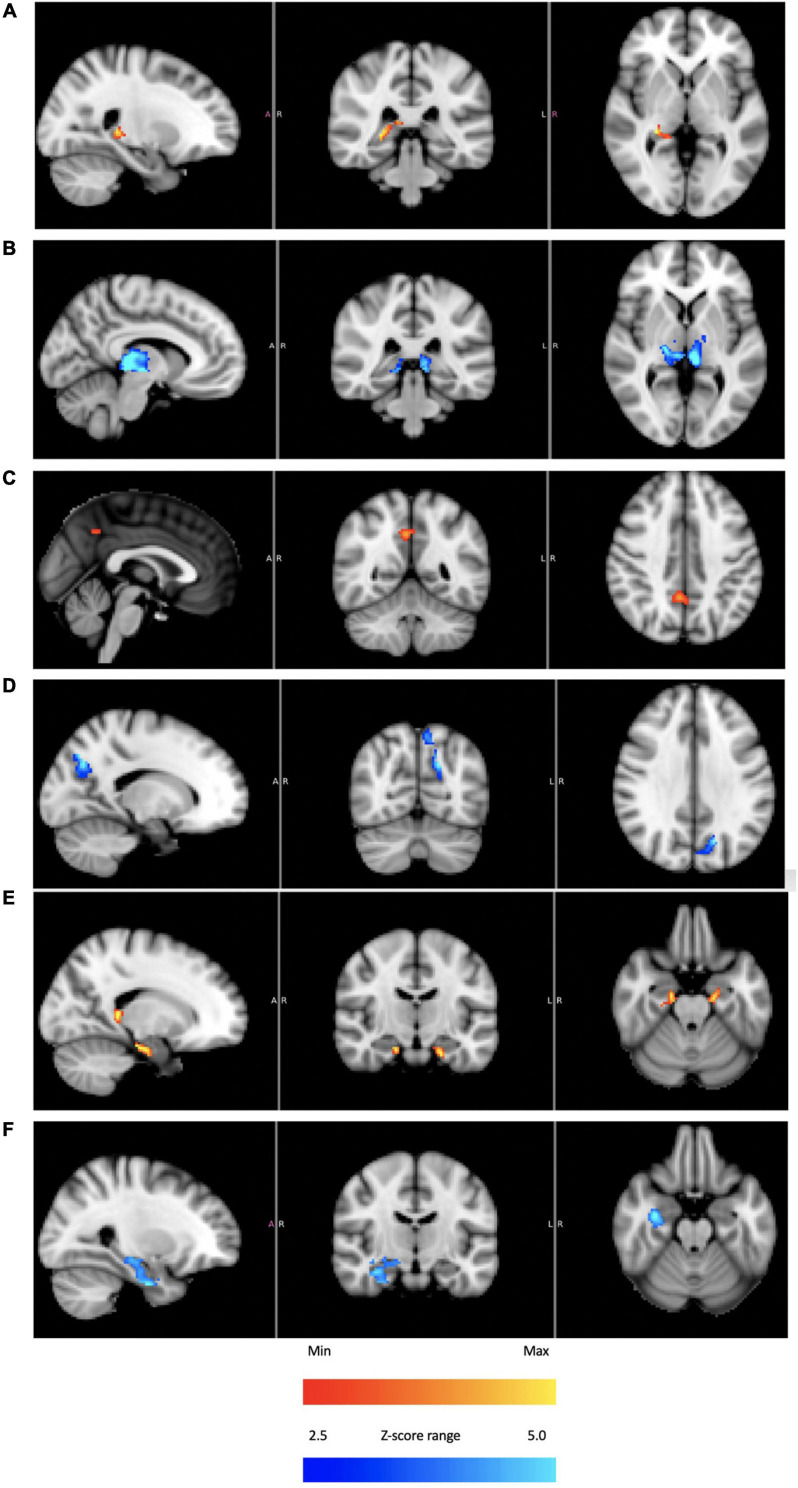
For subject 4: thalamus [**(A)** plateaus dominant, **(B)** troughs dominant]; precuneus [**(C)** plateaus dominant, **(D)** troughs dominant]; hippocampus [**(E)** plateaus dominant, **(F)** troughs dominant]. The color bars show relative z-score values. The orange/red and blue/aqua color bars are *not* related to each other.

Figure 8 provides initial evidence consistent with modulation of structures that process vestibular sensory input. Using subject 4 as an exemplar, BOLD responses from the thalamus, precuneous and hippocampus are shown. In all cases, plateaus dominant and troughs dominant images are included. Subjects 1–3 had corresponding responses, but as in the case of the cerebellar IC, these subjects did not have the highly coherent BOLD intensity recorded in subject 4.

**FIGURE 9:**
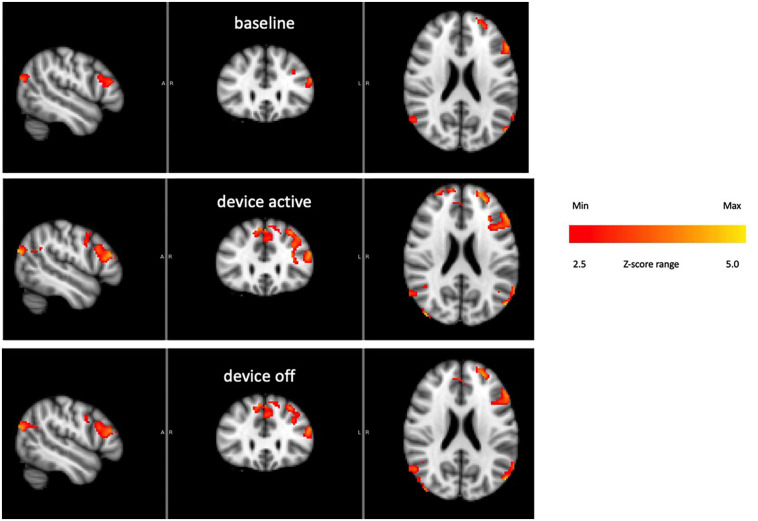
Frontoparietal network activity over the three phases of the BOLD run (all subjects). The color bar show relative z-score values.

**FIGURE 10:**
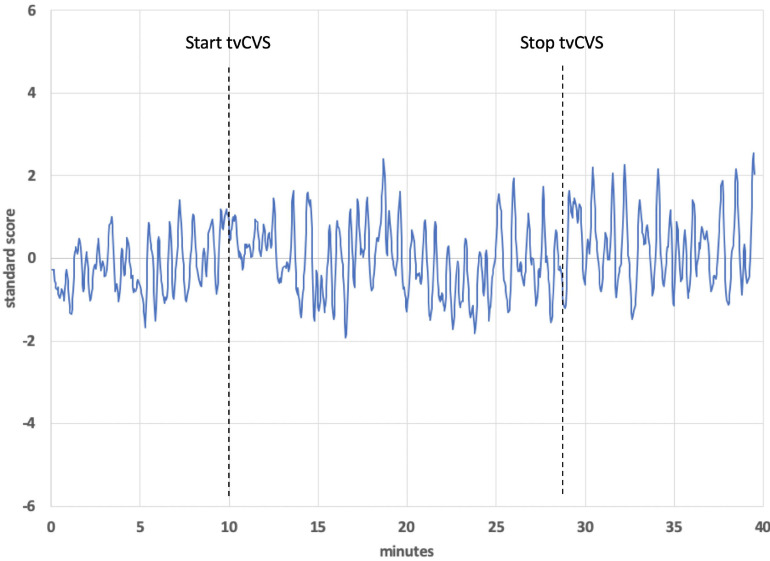
Time series from a frontoparietal independent component, subject 1. The dotted lines show the start and stop points of the tvCVS delivery.

Figure 9 shows the BOLD response identified by a block design in a frontoparietal network (averaging all subjects). Notice that the signal persists after tvCVS stimulation stops. This is unlike the previous independent components presented where the strongest modulation was seen during the device-active period. As can be seen in Figure 10, the frontoparietal independent component showed a buildup in intensity and a shift in spectral character that was delayed past the start of the device-active period and persisted through the device-off period.

### Assessment of Potential Artifacts

As part of the preprocessing pipeline for the BOLD data, a non-aggressive ICA-based Automatic Removal of Motion Artifacts (AROMA) was used to denoise the data (Pruim et al., 2015). This efficient software is very effective in estimating and eliminating artifactual data that may be caused by movements. We also utilized MELODIC v3.0 for the ICA analysis. This software decomposes the data into separate functional network and artifact components. One of us (RPB) has extensive experience using this methodology and was able to visually assess the components and classify them as either functional networks or artifacts. The components listed in the manuscript did not have any classical signatures associated with scanner or biologically produced artifacts. Each component is associated with some amount of variance from the overall BOLD signal. The ICA analysis splits the data into separate components, which are then assigned variance percentages, which describe the amount of variance of the original spatio-temporal maps. Specifically, this component accounted for 6.12% of explained variance (discrepancy between a model and actual data).

We also directly measured the electrical current output of the tvCVS device as it advanced through the generation of cold and warm waveforms to rule out artifacts. The temperature controller uses a pulse-width-modulation scheme that switches at 1.3 kHz. The polarity of the current supplied to the Peltier devices (one on each side of the headset) switches in order to keep the actual temperature matched to the target values. The current patterns switched many times during each temperature cycle. Therefore, the pattern of the current output, and the magnetic field it would generate, cannot explain the square wave shape of Figure 4, for example.

## Discussion and Conclusion

There have been previous imaging studies involving irrigation-based CVS (Karnath and Dieterich, 2006; Dieterich and Brandt, 2008; Lopez et al., 2012) and Klingner et al. (2013) used ICA to identify multiple networks that were affected by CVS. Time-varying CVS is fundamentally different from fixed temperature irrigations that are used diagnostically and that have been used in most previous CVS studies. For example, as discussed above, tvCVS showed evidence for the induction of CBFv oscillations, whereas constant temperature CVS did not induce oscillations. tvCVS is able to maintain consistent activity over time, unlike fixed temperature CVS that shows a decay in induced effects after a few minutes (Bock et al., 1979). The present work was motivated by a desire to understand how tvCVS might be enacted in an MRI environment and to produce preliminary BOLD data showing the effects of tvCVS on the brain so as to inform future imaging studies. Additionally, this study provided an opportunity to evaluate the concept of sensory neuromodulation (Black and Rogers, 2020) and how it might support future randomized controlled trials evaluating tvCVS therapy.

### Evidence for Thermoconvection

It is important to know how the tvCVS device induces changes in neuronal activity. The data collected in this study align with the dominant observed effects being consistent with the original Barany model. The thermoconvection model of CVS, first suggested by Barany and Wittmaack (1911), describes how endolymph in the segment of the horizontal semicircular canal along the inner ear wall, where heat transfer first occurs during CVS, can become buoyant relative to the more distal segment of the canal when a warm stimulus is applied. (During CVS induction, the subject is nearly supine so that the horizontal canal is actually in a vertical orientation. This acts to maximize the CVS induction effect.) The warmed endolymph becomes more buoyant and moves the cupula from its equilibrium position, resulting in an increased firing rate of hair cells above their equilibrium rate (of about 100 Hz). Cold CVS has the opposite effect, leading to a decreased firing rate. How accurately and completely the thermoconvection model describes the action of CVS has been debated and limited data from astronauts (Scherer and Clarke, 1985) seemed to demonstrate that the CVS phenomenon can occur in zero gravity, which is not consistent with the basic thermoconvection model (since “buoyancy” only makes sense in a gravitational field). Various alternative explanations have been summarized (Kassemi et al., 2005; Hood, 1989; McGeoch, 2010). Minor and Goldberg (1990) estimated that ∼75% of the effects of CVS result from thermoconvection. There is also evidence that hair cells other than those innervating the horizontal canal have altered firing rates during CVS (Tsuji et al., 1990, 1994; Shen et al., 2013). Our observations using ICA seem to support thermoconvection as the principle means for neuromodulation, as described below.

**FIGURE 11:**
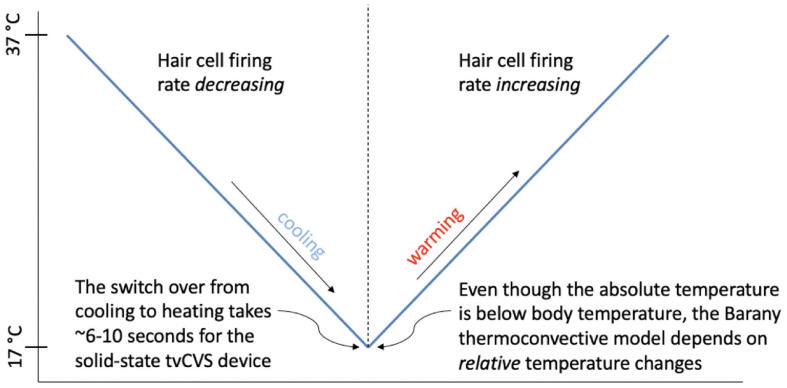
Depiction of the transition between hair cell firing rate states as a function of the direction of the temperature ramp.

Figure 4 shows the independent component located in the cerebellum (subject 4) superimposed with the applied thermal waveform. It is clear that the cold waveform period matches the period of the square wave, which represents the cerebellar independent component (the dotted lines in Figure 4A are included to emphasize the alignment). The ICA output was compared to known noise components to rule out alternative explanations for the ICA results. It is particularly noteworthy that the transition from the plateau to the trough of the independent component occurs over a very short time (<10 s) and corresponds to when the cold temperature ramp switches direction. To understand how this might occur, consider the earlier description of how endolymph becomes less buoyant when the inner ear wall is cooled below body temperature. It is less buoyant than fluid in the more distal segment of the canal, which is closer to body temperature. As the direction of the temperature ramp switches, to a warming ramp, the inner ear wall starts to become warm relative to the previously reduced temperature created by the cooling ramp (Figure 11). Therefore, the endolymph closest to the inner ear wall becomes warm relative to the previously cooled surrounds. Marcelli et al. (2009) provided evidence that changes in BOLD signal intensity occur within ∼5 s after delivery of a CVS stimulus. Therefore, the change in buoyancy in the horizontal semicircular canal should occur rapidly and the change in the conformation of the cupula should track, leading to a sharp change in the afferent firing rate of the vestibular hair cells innervating the horizontal canal. The timeframe of the change from a reduced firing (trough) rate to an increased one (plateau) is the time interval associated with the edge of the square wave as it transitions from a trough to a plateau. The y-axis of Figure 4, the standard score, is proportional to BOLD intensity and a plateau represents a higher firing rate of the afferent output of the hair cells and a trough is due to a reduced rate.

In order to explain Figure 4A, one must posit a mechanism that will produce a sharp square wave transition during a time when the change in applied temperature is on the order of 1°C or less: effectively a constant temperature in terms of thermally mediated processes. The thermoconvection model provides a ready explanation, versus models invoking a direct temperature effect on neuronal firing rates (such as from the trigeminal nerve) or structures in the inner ear (Hood, 1989; Bell, 2019), and demonstrates that the primary source of BOLD response in this study is consistent with a vestibular origin. The lateralized cerebellar contrast in Figure 5 may reflect the documented connectivity between the vestibular nuclei and the posterior cerebellum (Barmack, 2003).

We speculate that the additional structure seen in the “flat” regions of the plateaus and troughs in Figure 4B may be due in part to the modifying effects of the warm tvCVS waveform that is applied simultaneously to the contralateral ear. Dotted lines were added in Figure 4B to match features in the (warm) thermal waveform to corresponding points in the ICA trace. If the small changes in temperature are responsible for the noticeable changes in standard score (BOLD intensity) in Figure 4B, then the thermal responsivity of the vestibular organ (presumably the cupula in the horizontal semicircular canal) is quite high, which is consistent with reports in the literature (Peitersen, 1974).

That small changes in temperature may result in substantial changes in BOLD response is also evident in Figures 6, 7, showing a region in the operculum of subject 1. Once again, dotted lines were added to match features in thermal waveform (warm) to corresponding points in the ICA trace in Figure 7. The independent component located in the operculum tracks the period of the warm, not the cold, applied waveform, unlike in Figure 4A. It would not be unexpected for the applied stimulus to induce different resonant responses in different brain regions and in different individuals. We might speculate that the position of the cupula becomes metastable as a result of the time-varying thermal stimulus delivered by tvCVS. The ICA data from this study suggest the possibility that small deformations of the cupula away from its equilibrium position can result in a nearly binary switching between low firing and high firing states.

A notable observation from Figures 4, 7 is that the response in the cerebellum persists for the entire period the device is on (∼18.5 min) without diminution. Using constant temperature CVS, partial adaptation occurs within minutes (Bock et al., 1979). Time-varying CVS does not allow the vestibular hair cells to adapt, since the applied stimulus is constantly changing. Therefore, tvCVS enables longer duration run times when considering its use for therapeutic applications. The time rate of change of temperature is slow relative to irrigation CVS and this has been found to significantly reduce the induction of dizziness and nausea that can accompany the diagnostic CVS procedure.

Knowing that the fMRI signal analyzed in this study is consistent with a vestibular origin is an important starting point before considering activity in other sensory network hubs like the thalamus, hippocampus and precuneous. Below, we discuss how the broad activation seen in Figure 8 is consistent with the idea of sensory neuromodulation, which proposes that a modulatory signal that enters through a sensory organ is carried along the endogenous network innervated by that organ.

An important question is why subjects responded differently. Subject 4 exhibited a strong cerebellar response as seen in Figure 4 (correlated ICA signal) and Figure 5 (BOLD intensity relative to the other three subjects). Since we worked to eliminate artifacts in the data, results from subject 4 cannot be ignored, but neither can they be generalized without collecting data from additional subjects in the future. Subject 4 had exceptionally strong responses in the cerebellum; subject 1 showed a strong ICA response in the operculum (Figure 7); all four subjects showed similar BOLD responses in the frontoparietal network (Figure 9, discussed below). It is possible that these observations are signs of individualized responses in the context of this particular experimental protocol. It is also possible that responses in a single individual vary with thermal waveform choice and from day-to-day. The clinical data acquired using the thermal waveform of Figure 2 (Wilkinson et al., 2017, 2019) suggest that this waveform can be clinically effective in a broad population and so it would be a mistake to conclude that strong ICA signals, for example, are crucial for clinical utility. It may be that the periodic stimulation induced by these particular thermal waveforms creates a strong resonance in individuals who have natural network frequencies that are well matched to the applied stimulus (Black et al., 2016). It is well known that individuals show variability in the magnitude of response to standard CVS diagnostic protocols (Patki et al., 2016) and therefore some of the observed differences in this study may result from the underlying receptivity to CVS induction. We re-emphasize that the data in the current study should be viewed as proof-of-concept only and that significant work remains before making any firm conclusions about how a given subject responds acutely to tvCVS.

### Activity in Network Hubs

The innervation of the vestibular system is extensive (Kirsch et al., 2015) and one goal of the current study was to gather preliminary data on how different brain regions respond to tvCVS. There is a rich literature documenting the connectivity between the vestibular nuclei and the cerebellum (Rochefort et al., 2013; Hilber et al., 2019), the thalamus (Lopez and Blanke, 2011), the precuneous (Dieterich et al., 2003; Petit and Beauchamp, 2003; Falconer and Mast, 2012; Gottlich et al., 2014), and the hippocampus (Vitte et al., 1996; Smith, 1997; Cuthbert et al., 2000; Stackman et al., 2002; Smith et al., 2010; Tai and Leung, 2012; Hitier et al., 2014). The PIVC (parieto-insular vestibular cortex) is a multisensory convergence area that tends to be dominant in the non-dominant hemisphere (Dieterich et al., 2003) and it has been extensively studied in protocols involving artificial vestibular stimulation (Petit and Beauchamp, 2003; Ferrè et al., 2012; Frank and Greenlee, 2014; Shinder and Newlands, 2014).

As argued above, the applied thermal waveform effectively led to two different states: hair cells firing above the equilibrium rate or below it. That two-state behavior is reflected in BOLD response localized to the thalamus (Figures 8A,B), precuneous (Figures 8C,D), and hippocampus (Figures 8E,F). These regions are generally understood to be “rich club” areas (van den Heuvel and Sporns, 2011, 2013) that process and distribute sensory information to create a comprehensive representation of sensory perceptions. As was the case with the cerebellar independent component, the thalamus, hippocampus and precuneous showed activity immediately when the tvCVS stimulation started. It is not surprising to see evidence of coherent BOLD activity in these hub regions. These hubs are implicated in the anatomy of brain disorders (Crossley et al., 2014) and are therefore of particular interest.

### Frontoparietal Network

Recognizing that the results presented here are preliminary, nonetheless the fact that one brain region responds to one stimulus frequency and another region responds to a different (yet concurrent) frequency is noteworthy. This is the sort of finding one expects from the concept of sensory neuromodulation since the underpinning framework is that input to sensory organs is transformed as it travels over the endogenous sensory network. Such transformations allow brain regions remote from the sensory organs to properly interpret and act on the input stimulus. This is categorically different from neuromodulation methods that seek to impose an externally applied, common stimulus without regard to local neuronal dynamics (Danilov et al., 2015).

The BOLD time series for the frontoparietal independent component (Figure 9) does not follow the pattern found in the brain regions already discussed. For the cerebellum, thalamus, precuneous, hippocampus, and operculum, the modeled data showed increased activity as the stimulation period started and that activity effectively stopped when the stimulation ended. The frontoparietal independent component shows onset that is delayed with respect to the time stimulation starts and it persists into the post-stimulus period (Figure 10). In the earlier transcranial Doppler sonography study evaluating the effects of tvCVS (Black et al., 2016), the effects on CBFv also showed a delayed onset (after the start of tvCVS) and a persistent effect after the end of tvCVS. In that case, it was hypothesized that CBFv oscillations were due to the entrainment of a pontine pacing network that controls B wave activity. The delay in the onset of the CBFv response can be understood as resulting from time for entrainment of a pacing system that acts to modulate flow from the heart to the brain and back. The concept of neuronal entrainment and its role in brain function more generally has been recently summarized (Lakatos et al., 2019).

The frontoparietal independent component time series for each subject was analyzed using a fast Fourier transform algorithm (StatPlus, AnalystSoft, Inc.) in order to gain insight about the post-tvCVS activity, in particular. In all subjects, the principal spectral peaks in the post-tvCVS epoch represented periods of 50–85 s. Those values are consistent with blood circulation times (Morris et al., 2009). We hypothesize, therefore, that the spectral peaks represent evidence of the entrainment of blood flow that persisted after the end of the tvCVS stimulus, consistent with the conclusions reached in the transcranial Doppler study that evaluated tvCVS entrainment (Black et al., 2016). We posit that it is this persistent activity that accounts for the sustained contrast effects in Figure 9 (device-off). It is notable that the spectral power tends to show a narrow range of frequencies once the driving effect of tvCVS is removed, versus what can be a more complex admixture of frequencies when tvCVS, with two different frequency thermal waveforms that interact, is driving the BOLD activity. The frontoparietal network is removed from direct processing of incoming sensory information and this may be why its temporal response characteristics are different from those seen in the sensory processing areas.

### Neurovascular Coupling and Systemic Flow

In summary, the study results support several tentative propositions:

1 The principal means by which tvCVS modulates brain activity is consistent vestibular stimulation induced by thermoconductive effects and *not* by direct thermal effects on hair cells, other nerve bodies or non-neuronal structures. The small amount of data collected does not allow for a definitive conclusion on this point, however.
2 Brain regions involved in vestibular sensory processing respond primarily to the time-varying stimulus in the manner expected: the BOLD signal resulting from NVC. In particular, the modulatory effect of tvCVS occurs when the device is active and the alternations in the thermal waveforms drive BOLD contrast patterns.
3 In addition to NVC, there is evidence of a systemic blood flow effect that dominated the response characteristics in a frontoparietal network. That both NVC and systemic effects can change BOLD contrast has been established (Tong et al., 2019).
4 The fMRI results are consistent with the concept of sensory neuromodulation since there is evidence that different brain regions respond differently to the tvCVS stimulus, reflecting a transformation of the incoming stimulus pattern as it propagates through the vestibular sensory network.

We have provided evidence for the modulation of cerebral blood flow, both via NVC and systemic flow effects, by tvCVS and we have reviewed the critical importance of hemodynamics in maintaining brain health and function. Multiple forms of vestibular neuromodulation (tilt/rotation, sinusoidal GVS, tvCVS) have been used to induce low frequency oscillations in hemodynamic variables. We are not aware of other neuromodulation device studies that produce such effects. However, the exercise literature, in particular studies of high intensity interval training, does contain examples of time-varying modulation of hemodynamics (Klein et al., 2019). The well-known benefits of aerobic exercise on brain health (Barnes, 2015) makes it an interesting model for comparison as we address future clinical work utilizing tvCVS.

### Conclusions, Limitations, and Future Work

This work is limited in terms of enabling conclusive statements about the acute effects of tvCVS on BOLD contrast in multiple brain regions. Our primary purpose was to provide a proof-of-concept demonstration that tvCVS can be performed in a fMRI imaging experiment. A short listing of questions that remain unanswered:

1 Why did subject 4 have such a large cerebellar response and subject 1 have a large response in the operculum? We hypothesize that the answer lies in a resonant response of a “metastable cupula,” a proposal that is bolstered by the apparent sensitivity to small temperature changes evident in Figures 4, 7. All subjects showed comparable BOLD effect sizes in the frontoparietal network data, suggesting robust CVS induction in all subjects.
2 Why do the plateaus and troughs, e.g., in Figure 4, lead to different BOLD contrast in Figure 5? Presumably this reflects the manner in which the brain stem and cerebellum process changes in the afferent firing rate from the horizontal semicircular canal.
3 More generally, what gives rise to the lateralized effects seen in the ICA-delineated BOLD images? The applied tvCVS waveforms are inherently lateralized, of course. But if the BOLD contrast seen in Figure 9 is due to a systemic blood flow effect, why does there seem to be a left-sided dominance? This may be due to an inherent bias in activity in the network for right-handed subjects.
4 How might these results be interpreted for the purpose of titrating tvCVS in a therapeutic context if, for example, the frequency of the stimulus waveform plays a role in the strength of response? Future work must evaluate the effects of changing the laterality and frequency of thermal stimulation waveforms.
5 It is beyond the scope of this report, but tvCVS is a potentially interesting probe of resting state brain function since it can act in the low-frequency regime commonly used to study the default mode network (Raichle, 2015; Demertzi et al., 2019) and it is a task-neutral perturbation.

The widespread brain responses to tvCVS, especially in the frontoparietal network, is interesting in terms of current theories about vestibular cognition (Smith, 2017; Ferrè and Haggard, 2020). Clinically, the widespread activation patterns are potentially meaningful in explicating recent tvCVS clinical results that show evidence of broad efficacy across symptoms that involve dysfunction in multiple brain networks (Wilkinson et al., 2019). Vestibular neuromodulation approaches including rotary chairs (Serrador et al., 2009), sinusoidal GVS (Cohen et al., 2011) and tvCVS (Black et al., 2016) have all demonstrated the ability to induce oscillations in hemodynamic parameters and this is a capability that has not been demonstrated with other neuromodulation approaches. Hemodynamic modulation informs current hypotheses of the mechanism of action of tvCVS in a therapeutic context (Black and Rogers, 2020).

## Data Availability Statement

The raw data supporting the conclusions of this article will be made available by the authors, without undue reservation.

## Ethics Statement

The studies involving human participants were reviewed and approved by Wake Forest Baptist Medical Center, Wake Forest, NC, United States. The patients/participants provided their written informed consent to participate in this study.

## Author Contributions

RDB: primary author, corresponding author, and primary interpretation of results. RPB: secondary author and image reconstruction and analysis. KR and CS: editor for vestibular neuroscience content. RP: built and tested the vestibular neuromodulation device. CL: editor for radiological interpretation. CW: editor for radiological interpretation and designed fMRI scan sequence. All authors contributed to the article and approved the submitted version.

## Conflict of Interest

RDB and RP were employed by company Scion NeuroStim, LLC. The remaining authors declare that the research was conducted in the absence of any commercial or financial relationships that could be construed as a potential conflict of interest.

## Publisher’s Note

All claims expressed in this article are solely those of the authors and do not necessarily represent those of their affiliated organizations, or those of the publisher, the editors and the reviewers. Any product that may be evaluated in this article, or claim that may be made by its manufacturer, is not guaranteed or endorsed by the publisher.
