# Supplementary material for: The Acute Effects of Time-Varying Caloric Vestibular Stimulation as Assessed With fMRI
Source: Front Syst Neurosci. 2021 Aug 9;15:648928. doi: 10.3389/fnsys.2021.648928 (PMC8381736; doi:10.3389/fnsys.2021.648928)
Supplement: Supplementary file 1 [file Data_Sheet_1.docx]

**Supplemental Section**

fMRI data preprocessing:

The data were preprocessed with version stable fMRIPrep (Esteban et al., 2019), which utilizes tools from multiple software packages. FMRIPrep is a Nipype based tool (Gorgolewski et al., 2011), which also uses Nilearn (Abraham et al., 2014). Each T1w volume was corrected for intensity non-uniformity using N4BiasFieldCorrection (v2.1.0; Tustison et al., 2010) and skull-stripped using ANTs (v5.0.9; Avants et al., 2008). Freesurfer was used to reconstruct brain surfaces (v6.0.1; Dale et al., 1999), which were then used to refine the skull-stripping (Klein et al., 2017). The T1w volume was spatially normalized to MNI space using nonlinear registration in ANTs (Avants et al., 2008; Fonov et al., 2009). Brain tissue segmentation of cerebrospinal fluid, white-matter and gray-matter was also performed using FAST (FSL v5.0.9; Zhang et al., 2001).

The first six timepoints were discarded from the functional data to allow the scanner to reach a steady state. Functional data were slice-time-corrected using AFNI (v16.2.07; Cox, 1996) and motion-corrected using FSL’s MCFLIRT (Jenkinson et al., 2002). "Fieldmap-less" distortion correction was performed by co-registering the functional image to the same-subject, intensity-inverted T1w structural image, constrained with an average fieldmap template (ANTs; Huntenburg, 2014; Treiber et al., 2016). This was followed by co-registration to the T1w image using boundary-based registration in Freesurfer (Greve & Fischl, 2009). Motion correction, field distortion correction, co-registration, and normalization were applied in a single step using Lanczos interpolation using ANTs. Non-aggressive ICA-based Automatic Removal of Motion Artifacts (AROMA) was used to denoise the data (Pruim et al., 2015). Functional images were then subject to high-pass filtering using FSL with a cutoff of 100 seconds.

Generation of plateau/trough dominant images:

Individual time-series statistical analysis was carried out using FILM with local autocorrelation correction. The blocks were defined as the plateau and trough periods during the intervention period and entered as regressors that were then convolved with a double-gamma hemodynamic response function. Contrasts were defined as plateaus > troughs and plateaus < troughs. Statistical contrasts of the blocks were conducted using a fixed-effects analysis. For visualization, task activation maps for all participants combined were generated using FSL’s Local Analysis of Mixed Effects (FLAME 1+2) with a cluster threshold of Z >2.6 and p <.05, corrected over the entire brain using Gaussian random field theory (Woolrich et al., 2001).

Block design:

Individual time-series statistical analysis was carried out using FILM with local autocorrelation correction. The blocks were defined as pre-intervention, intervention, and post-intervention, and entered as regressors that were then convolved with a double-gamma hemodynamic response function. Statistical contrasts of the blocks were conducted using a fixed-effects analysis. For visualization, task activation maps for all participants combined were generated using FSL’s Local Analysis of Mixed Effects (FLAME 1+2) with a cluster threshold of Z >2.6 and p <.05, corrected over the entire brain using Gaussian random field theory (Woolrich et al., 2001).
